# Congenital polymorphic cataract associated with a G to A splice site mutation in the human beta-crystallin gene *CRYβA3/A1*

**Published:** 2012-08-08

**Authors:** Yibo Yu, Jinyu Li, Jia Xu, Qiwei Wang, Yinhui Yu, Ke Yao

**Affiliations:** Eye Center of the 2nd Affiliated Hospital, Medical College of Zhejiang University, Hangzhou, China

## Abstract

**Purpose:**

To identify the underlying genetic defect in four generations of a Chinese family affected with bilateral congenital polymorphic cataracts.

**Methods:**

Family history and clinical data were recorded. The phenotype was documented using slit-lamp photography. Genomic DNA samples were extracted from peripheral blood of family members. Candidate genes were amplified using polymerase chain reaction (PCR) and screened for mutations on both strands using bidirectional sequencing.

**Results:**

Affected individuals exhibited variable opacities in the embryonic nucleus, sutures, and peripheral cortical opacities. The phenotype for this family was identified as polymorphic. Direct sequencing revealed a splice site mutation (c.215+1G>A) at the first base of intron 3 of the crystallin beta A3/A1 (*CRYBA3/A1*) gene. This mutation co-segregated with all affected individuals in the family and was not found in unaffected family members or in 100 unrelated controls.

**Conclusions:**

Our results identified a recurrent c.215+1G>A mutation in *CRYBA3/A1* in a polymorphic congenital cataract family, summarized the variable phenotypes among the patients, which expanded the phenotypic spectrum of congenital cataract in a different ethnic background, and suggested a mechanism that influences cataractogenesis.

## Introduction

Congenital cataract, the loss of eye lens transparency, is a significant cause of visual impairment or blindness in childhood. The prevalence of congenital cataracts is 1 to 6 per 10,000 live births, depending on the ascertainment method [1]. Globally, congenital cataracts account for nearly one-tenth of childhood blindness from different causes including infections during embryogenesis, metabolic disorders (galactosemia), and genetic defects [2].Statistical analyses have revealed that about one quarter of congenital cataracts are hereditary [3].Genetically, the majority of isolated congenital cataracts exhibit as autosomal dominant, although autosomal recessive and X-linked inherited forms have also been reported [4].

Over the past few years, remarkable progress has been made toward our understanding of the cataractogenesis process. Currently, there are more than 40 genetic loci to which isolated or primary cataracts have been mapped, and more than 26 genes have been characterized, although this number is constantly increasing [5]. Autosomal dominant congenital cataracts (ADCC) was reportedly caused by mutations in different genes [2]. Approximately half of the mutations are in the crystallin genes and a quarter in connexin genes, with the remainder divided among genes that encode heat shock transcription factor-4 (*HSF4*), aquaporin-0 (*AQP0, MIP*), paired-like homeodomain 3 (*PITX3*), v-maf musculoaponeurotic fibrosarcoma oncogene homolog (*MAF*), chromatin modifying protein (*CHMP4B*), lens intrinsic membrane protein 2 (*LIM2*), beaded filament structural protein-2 (*BFSP2*), and other genes [2,6]. The crystallin and connexin genes appear to be the most commonly associated with congenital cataract. So, it is suitable to consider these genes as the top candidates for developing congenital cataracts screening strategies.

Congenital cataracts can be classified into several subtypes according to morphology: total, nuclear, cortical, anterior polar, posterior polar, lamellar, cerulean, pulverulent, sutural, coralliform, wedge-shaped, and polymorphic cataracts and other minor subtypes [2].Congenital cataracts are genetically heterogeneous [7]. It is known that different mutations in different genes can cause similar cataract patterns, while the highly variable cataract morphologies within some families suggest that the same mutation in a single gene can lead to different phenotypes [8,9].

In this paper, a four-generation family affected with congenital polymorphic cataracts was investigated in an attempt to identify the genetic defect associated with their cataract phenotype.

## Methods

### Clinical evaluations and DNA specimens

**Figure 1:**
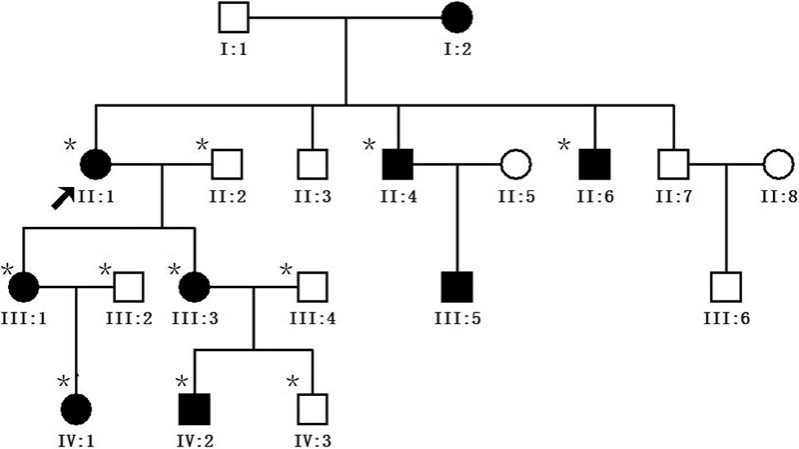
Pedigree of the autosomal dominant congenital cataract mutation. The proband is marked with an arrow. Squares and circles indicate males and females, respectively. Black and white symbols represent affected and unaffected individuals, respectively. The asterisks indicate family members who attend this study.

**Figure 2:**
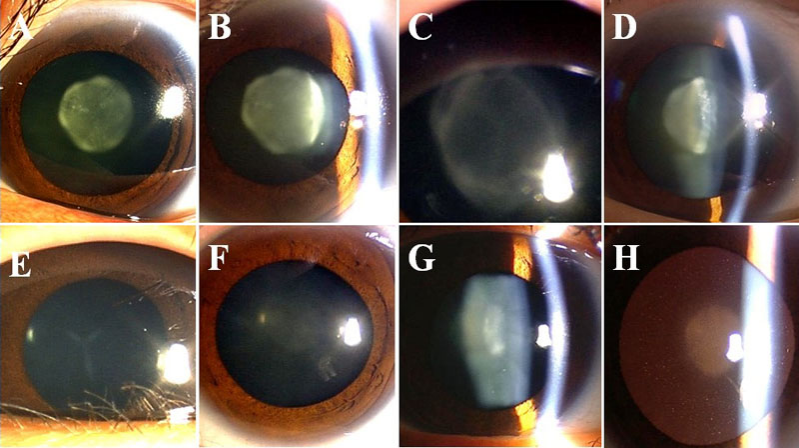
Slit-lamp photograph of family members with congenital cataracts. **A**, **B**: The proband (II:1) had nuclear cataract with 'Y' sutural opacities. **C**, **D**: The affected member IV:2 showed a different zonular cataract with 'Y' sutural opacities. **E**: The affected member IV:1 had simple 'Y' sutural opacities. **F**-**H**: The affected member III:3 had slight nuclear cataract with curd-like peripheral cortical opacities.

Four generations of a family suffering with ADCC were recruited from the Eye Center of Affiliated Second Hospital, College of Medicine, Zhejiang University, Hangzhou, China. Informed consent was obtained from all participants in accordance with the Zhejiang Institutional Review Board and the study protocol adhered to the tenets of the Declaration of Helsinki. In total, 11 individuals participated: 7 affected and 4 unaffected (Figure 1). Detailed medical histories were obtained by interviewing all individuals. All participants underwent detailed ophthalmic examinations including visual acuity, slit lamp examination with dilated pupils, ultrasonography, fundus exam, and intraocular pressure measurement. The phenotypes were documented using slit lamp photography (Figure 2). Also,100 unrelated ethnically-matched controls with no family history of congenital cataracts were recruited.

About 2 ml of peripheral blood was collected from the family members and the controls who took part in the study. Blood samples were obtained by venipuncture, collected in Vacutainer tubes (Becton-Dickinson, Franklin Lakes, NJ) containing ethylene diamine tetraacetic acid (EDTA). Leukocyte genomic DNA was extracted using the QIAmp Blood kit (Qiagen, Duesseldorf, Germany).

### Mutation analysis

Genomic DNA samples from affected and unaffected members of the family were screened for mutations in crystallin alpha A (*CRYAA*), crystallin alpha B (*CRYAB*), crystallin beta A3/A1 (*CRYBA3/1*), crystallin beta B2 (*CRYBB2*), crystallin gamma C (*CRYGC*), crystallin gamma D (*CRYGD*), gap junction protein, alpha 3 (*GJA3*), and gap junction protein, alpha 8 (*GJA8*) genes using direct sequencing. The coding regions of candidate genes were amplified using polymerase chain reaction (PCR) with previously published primer sequences (Table 1) [10-17]. The cycling conditions for PCR were 95 °C pre-activation for 5 min, 10 cycles of touchdown PCR with a 0.5 °C down per 60 °C to 55 °C cycle, followed by 30 cycles with denaturation at 95 °C for 25 s, annealing at 55 °C for 25 s, and extension at 72 °C for 40 s. PCR products were isolated using electrophoresis on 3% agarose gels and sequenced using the BigDye Terminator Cycle sequencing kit V 3.1 (ABI–Applied Biosystems; Sangon Co, China) on an ABI PRISM 3730 Sequence Analyzer (ABI), according to the manufacturer’s instructions. Sequencing results were analyzed using Chromas 1.62 and compared with sequences from NCBI GenBank (*CRYAA*: 21q22.3; NM_000394, *CRYAB*: 11q22; NG_009824, *CRYBA1*: 17q11-q12; NM_005208, *CRYBB2*: 22q11.2; NM_000496, *CRYGC*: 2q33-q35; NM_020989, *CRYGD*: 2q33-q35; NM_006891.3, *GJA3:* 13q11-q13; NM_021954, and *GJA8:* 1q21-q25; NM_005267).Direct sequencing was also used to screen the mutation identified in *CRYBA1*on 100 ethnically-matched controls to confirm the mutation.

**Table 1:** Polymerase chain reaction primers and product sizes.

**Name**	**Primer sequence (5′-3′)**	**Product size (bp)**
***CRYBA3/1***		
Exon-1 F	5′GGCAGAGGGAGAGCAGAGTG 3′	207
Exon-1 R	5′CACTAGGCAGGAGAACTGGG 3′	
Exon-2 F	5′AGTGAGCAGCAGAGCCAGAA 3′	293
Exon-2 R	5′GGTCAGTCACTGCCTTATGG 3′	
Exon-3 F	5′AAGCACAGAGTCAGACTGAAGT 3′	269
Exon-3 R	5′CCCCTGTCTGAAGGGACCTG 3′	
Exon-4 F	5′GTACAGCTCTACTGGGATTG 3′	357
Exon-4 R	5′ACTGATGATAAATAGCATGAACT 3′	
Exon-5 F	5′GAATGATAGCCATAGCACTAG 3′	290
Exon-5 R	5′TACCGATACGTATGAAATCTGA 3′	
Exon-6 F	5′CATCTCATACCATTGTGTTGAG 3′	295
Exon-6 R	5′GCAAGGTCTCATGCTTGAGG 3′	
***CRYAA***		
Exon-1 F	5′CTTAATGCCTCCATTCTGCT 3′	593
Exon-1 R	5′TGGCTGGTGCCTTACAAA 3′	
Exon-2 F	5′ CACCTGACCATAGCCAAACAAC 3′	512
Exon-2 R	5′ TCTCCCAGGGTTGAAGGCA 3′	
Exon-3 F	5′ GGGGCATGAATCCATAAATC 3′	487
Exon-3 R	5′ GGAAGCAAAGGAAGACAGACAC 3′	
***CRYAB***		
Exon-1 F	5′ AACCCCTGACATCACCATTC 3′	469
Exon-1 R	5′ GGAGGAAGGCACTAGCAACC 3′	
Exon-2 F	5′ TGCAGAATAAGACAGCACCTG 3′	296
Exon-2 R	5′ AATGTAGCCAGCCTCCAAAG 3′	
Exon-3 F	5′ TCTGCCTCTTTCCTCATT 3′	473
Exon-3 R	5′ CCTTGGAGCCCTCTAAAT 3′	
***CRYBB2***		
Exon-2 F	5′ TGCTCTCTTTCTTTGAGTAGACCTC 3′	385
Exon-2 R	5′CCCATTTTACAGAAGGGCAAC 3′	
Exon-3 F	5′ ACCCTTCAGCATCCTTTG G 3′	314
Exon-3 R	5′ GCAGACAGGAGCAAGGGTAG 3′	
Exon-4 F	5′ GCTTGGAGTGGAACTGACCTG 3′	244
Exon-4 R	5′ GGCAGAGAGAGAAAGTAGGATGATG 3′	
Exon-5 F	5′ GCCCCCTCACCCATACTC 3′	242
Exon-5 R	5′ CCCCAGAGTCTCAGTTTCCTG 3′	
Exon-6 F	5′ CCTAGTGGCTTATGGATGCTC 3′	347
Exon-6 R	5′ TCTTCACTTGGAGGTCTGGAG 3′	
***CRYGC***		
Exon-1.2 F	5′ TGCATAAAATCCCCTTACCGCTGA 3′	524
Exon-1.2 R	5′ ACTCTGGCGGCATGATGGAAATC 3′	
Exon-3 F	5′AGACTCATTTGCTTTTTTCCATCCTTCTTTC 3′	407
Exon-3 R	5′GAAAGAATGACAGAAGTCAGCAATTGCC 3′	
***CRYGD***		
Exon-1.2 F	5′ CCTCGCCTTGTCCCGC 3′	340
Exon-1.2 R	5′ TTAACTTTTGCTTGAAACCATCCA 3′	
Exon-3 F	5′ TGCTTTTCTTCTCTTTTTATTTCTGGGTCC 3′	400
Exon-3 R	5′AGTAAAGAAAGACACAAGCAAATCAGTGCC 3′	
***GJA3***		
Exon-1–1 F	5′ CTCTTCTGGCTCTGGCTTCC 3′	741
Exon-1–1R	5′ CACCTCGAACAGCGTCTTGA 3′	
Exon-1–2 F	5′ CTTCCCCATCTCCCACATCC 3′	749
Exon-1–2 R	5′ GGTGGCCGTTGTAGAGCTTG 3′	
Exon-1–3 F	5′ TCCGCCAAGCTCTACAACG 3′	535
Exon-1–3 R	5′ GAAACCTGATCTCTCCTCCAT 3′	
***GJA8***		
Exon-2–1 F	5′ CAGATATTGACTCAGGGTTG 3′	542
Exon-2–1R	5′ GATGATGTGGCAGATGTAGG 3′	
Exon-2–2 F	5′ GGCAGCAAAGGCACTAAG 3′	465
Exon-2–2 R	5′ CTCCACCATCCCAACCTC 3′	
Exon-2–3 F	5′ ATCGTTTCCCACTATTTCC 3′	492
Exon-2–3 R	5′ GGCGTCACTTCATACGGTTA 3′	

## Results

### Clinical evaluations

The cataract exhibited an autosomal dominant inheritance pattern in the family (Figure 1).Three of the seven patients had undergone lens surgery. All affected patients had bilateral lens opacification, but the degree of lens opacities was highly variable (Figure 2).The proband (II:1), who was a 59-year-old woman, had nuclear cataract with ‘Y’ sutural opacities (Figure 2A,B).The affected member III:3 (Figure 2F-H), who was the daughter of the proband, had slight nuclear cataract with curd-like peripheral cortical opacities, while her son (IV:2; Figure 2C,D) showed a different zonular cataract with ‘Y’ sutural opacities. The affected member IV:1(Figure 2E) had a simple ‘Y’ sutural opacity. The clinical evaluation of the affected individuals is provided in Table 2. Prior to surgery, the affected members had visual acuity ranging from 0.05 to 0.8. After surgery, all patients achieved a best-corrected visual acuity of 0.8 to 1.0. There was no family history of other ocular or systemic abnormalities.

**Table 2:** Clinical features of affected individuals.

**Affected individual**	**Gender**	**Age**	**Surgery age**	**Phenotype**
II:1	Female	59	59	Nuclear cataract with ‘Y’ sutural opacities
II:4	Male	55	43	IOL, after cataract surgery
II:6	Male	53	41	IOL, after cataract surgery
III:1	Female	34	29	IOL, after cataract surgery
III:3	Female	33	No surgery	Nuclear cataract with curd-like peripheral cortical opacities
IV:1	Female	6	No surgery	‘Y’ sutural opacities
IV:2	Male	8	8	zonular cataract with ‘Y’ sutural opacities and peripheral cortical opacities

### Mutation screening

**Figure 3:**
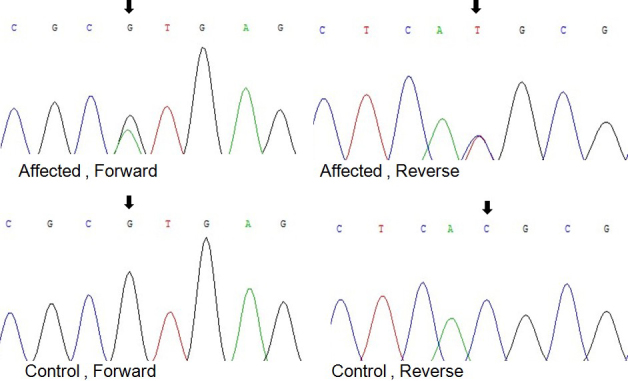
Forward and reverse sequence analyses of the affected and unaffected individuals in the ADCC Chinese family, showing a c.215+1G>A mutation of *CRYBA3/A1* (black arrows).

Through bidirectional sequencing of the coding regions of the candidate genes, we identified a c.215+1G>A substitution in the donor splice site of intron 3 in *CRYBA3/A1* in all affected individuals (Figure 3) that co-segregated with all affected individuals, whereas this heterozygous mutation was not present in the unaffected family members, nor in 100 unrelated Chinese without cataracts who served as controls.

## Discussion

In this study, we identified a splice site mutation within *CRYBA3/A1* in a four-generation Chinese pedigree with autosomal dominant polymorphic cataract.

Crystallins are known to constitute about 90% of the water-soluble proteins of the lens and contribute to transparency and refractive properties by forming a uniform concentration gradient in the lens. A mutation in the crystallin gene may alter crystallin stability, solubility, or ability to oligomerize and may precipitate from solution, resulting in lens opacity. So, they are considered to be good candidate genes for congenital cataract [18].The vertebrate crystallins are divided into two families: α-crystallins and the β- and γ-crystallin families [19,20]. The β- and γ-crystallins share a commonly features anti-parallel β-sheets in the proteins, referred to as the “Greek key motif.” All vertebrate lens β-crystallins consist of two domains and each one folds into two similar “Greek key motifs,” with each “Greek key motif” comprised of four consecutive anti-parallel β-strands [21].

The *CRYBA3/A1* gene uses an alternative translation initiation site to encode both the βA3- and βA1-crystallins.The βA3-crystallins are longer than the βA1-crystallins by the addition of 17 amino acids at the 5′-terminal end [22]. An intermediate form of the βA3-crystallin gene has an N-terminal arm shortened by 8 amino acids [23]. The βA1-crystallin aggregates ranged from dimers to octamers and further complexity is related to temporal and spatial regulation of expression as well as posttranslational modifications [24].

The *CRY*B*A3/A1* gene consists of six exons: the first two exons encode the N-terminal arm, and the subsequent four exons are responsible for the Greek key motifs [25]. So far, four mutations within the *CRYBA3/A1* gene was reportedly associated with congenital cataract in different families (Table 3).One is the c.215+1G>A mutation which we reported here, another is the c.215+1G>C [26], the third type is c.215+1G>T [27],and the fourth is a 3-bp deletion at positions 279–281 (c.279_281del) in exon 4, which causes an in-frame deletion of a glycine residue at position 91 (p.Gly91del) [28-31].

**Table 3:** Previous *CRYBA3/BA1* gene mutations associated with congenital cataracts.

**Bp exchange**	**Aa exchange**	**Biologic consequence**	**Origin of family**	**Reference**
c.215+1G>A	Splice site mutation	zonular lamellar opacities cataract and floriform	Indian	[6]
c.215+1G>C	Splice site mutation	pulverulent, star-shaped, shieldlike and radial cataract	Brazilian	[26]
c.215+1G>T	Splice site mutation	Y-suture, nucleus and cortical cataract	Chinese	[27]
c.215+1G>A	Splice site mutation	Y-sutural,mild nucleus and cortical dot cataract	Australian	[32]
c.215+1G>A	Splice site mutation	progressive childhood nucleus and peripheral cortex cataract	Chinese	[33]
c.215+1G>A	Splice site mutation	posterior polar cataract	Chinese	[34]
c.215+1G>A	Splice site mutation	zonular cataract with sutural opacity	Indian	[35]
c.279_281del	p.Gly91del	nuclear cataract	Chinese	[28]
c.279_281del	p.Gly91del	pulverulent nuclear congenital cataracts	Chinese	[29]
c.279_281del	p.Gly91del	pulverulent lamellar congenital cataracts	Chinese	[29]
c.279_281del	p.Gly91del	nuclear cataract	Swiss	[30]
c.279_281del	p.Gly91del	lamellar cataract	Britain	[31]

Previously, five geographically distinct families have been reported to possess the c.215+1G>A mutation, which is associated with diverse phenotypes including zonular, lamellar, nuclear, cortical,sutural, and posterior polar cataract [6,32-35].Diverse cataract phenotypes caused by exactly the same mutation within *CRYBA3/A1* in different ethnic backgrounds suggest that ethic background including environmental factors or, more likely, other genetic modifiers may influence the expression and function of this gene in lens development and cataract formation. In the family we studied, the phenotypes show considerable variation in morphology, and the severity of the disease ranged from requiring surgery to unawareness of the affliction before this study. Of the four patients who had pictures of their affected eyes taken,II:1 (nuclear cataract with ‘Y’ sutural) and IV:2 (zonular cataract with ‘Y’ sutural), are more severe than III:3 (mild nuclear cataract) and IV:1 (simple ‘Y’ sutural cataract). In addition, after a 5-year followed up of this family, we found the opacities of lens in the affected individuals are not progressive. So, the phenotype of this family was identified as polymorphic.Splice-site mutation is a genetic mutation that inserts or deletes several nucleotides at the splice junction during mRNA processing. It was reported to contribute to exon skipping, activation of cryptic splice sites, creation of pseudo-exon within an intron, or intron retention, which commonly results in exon skipping [36].As speculated by Kannabiran et al. [35], the c.215+1G>A mutation (position 474) would result in skipping of a donor splice junction, recruitment of a cryptic splice site (position 460),or possibly both. All possibilities would cause improper folding of the first Greek key motif, which leads to structural instability of βA1/A3-crystallin and subsequent cataract formation.

### Conclusions

In conclusion, we have identified a polymorphic form of congenital cataracts associated with a c.215+1G>A mutation of the *CRYBA1/A3* gene in a Chinese family. This mutation supports the role of the *CRYBA3/A1* gene in human cataract formation and provides additional evidence for the genetic heterogeneity of congenital cataracts in a different ethnic background.
